# Investigation of Resistance Switching and Synaptic Properties of VO_*x*_ for Neuromorphic Applications

**DOI:** 10.1021/acsomega.4c02001

**Published:** 2024-06-05

**Authors:** Gökhan Ekinci, Bünyamin Özkal, Sinan Kazan

**Affiliations:** † Department of Physics, Gebze Technical University, Kocaeli 41400, Türkiye; ‡ Department of Physics, Pîrî Reis University, Istanbul 34940, Türkiye

## Abstract

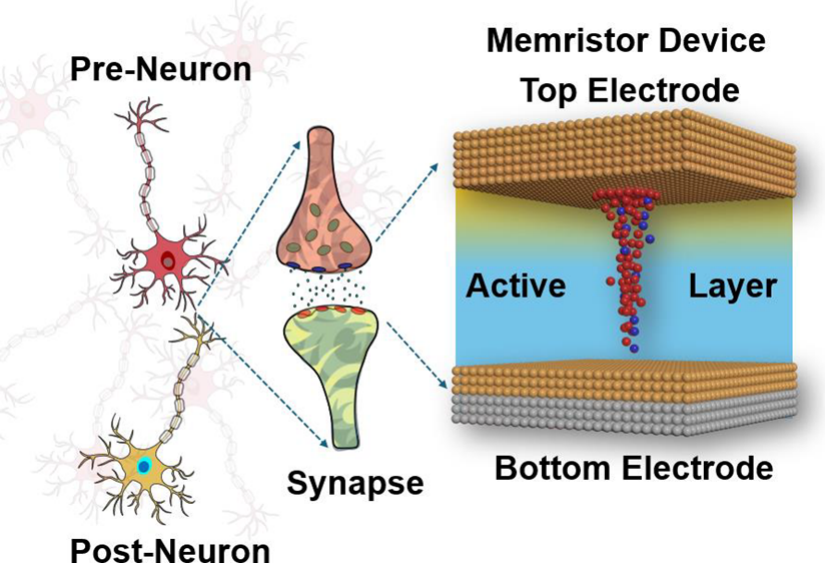

The taking run on artificial intelligence in the last decades is based on the von Neumann architecture where memory and computation units are separately located from each other. This configuration causes a large amount of energy and time to be dissipated during data transfer between these two units, in contrast to synapses in biological neurons. A new paradigm has been proposed inspired by biological neurons in human brains, known as neuromorphic computing. Due to the unusual current–voltage characteristic of memristor devices such as pinched hysteresis loops, memristors are considered a key element of neuromorphic architecture. In this study, we report the basic current–voltage characteristic of the memristor devices in the form of Si/SiO_2_/Pt(30 nm)/VO_*x*_ (3, 13, 25 nm)/Pt (30 nm) sandwich structure. Synaptic functions such as spike-time-dependent plasticity (STDP), paired-pulse facilitation (PPF), long-term potentiation (LTP), and long-term depression (LTD) of memristor devices were examined in detail. The oxide layer VO_*x*_ has been grown by using the VO_2_ target in a pulsed laser deposition (PLD) chamber. The composition and oxidation states of the oxide layer were examined using the X-ray photoelectron spectroscopy (XPS) technique. The status of oxygen vacancies, which play an active role in the operation of the devices, was examined with a photoluminescence (PL) technique. The experimental results showed that the thickness of the oxide layer can significantly influence the synaptic and resistive switching properties of the devices.

## 1 Introduction

Brain-inspired neuromorphic computing is a promising research field for artificial intelligence with efficient computing and low energy consumption.^1−8^ Currently, von Neumann’s architecture based on metal oxide semiconductor (CMOS) technology has been limited with the transistor dimension according to Moore’s law, stating that the number of transistors in integrated circuits will steadily increase every year.^9^ Moreover, an ever-increasing amount of transferring data such as the Internet of Things (IoT), and processing of these data requires more and more energy, scalability, and efficiency. Although digital computers are available today that can provide analogous approaches to the brain functionality of animals such as mice and cats, these lead to exponential energy consumption due to the high complexity of the brain functions of biological creatures.^10,11^ Because of all of this, extensive research continues vigorously to reveal new technologies and alternatives. Unlike the von Neumann architecture, brain neural networks are capable of event-driven, parallel, and nonlinear information processing. These neural networks, which also can process distributed information structures, demonstrate significant advantages in fault tolerance and power efficiency in vital functions such as voice recognition, movement control, reasoning, judgment, and high-density learning.^12−14^ Researchers focus on constructing information processing systems that can mimic the function of the biological nervous system by analogy to the working mechanism of the human brain. The human nervous system contains a vast number of synapses, up to 10^14^. If you aim to build a similar system by analogy, then you must consider the challenge of implementing a massively parallel system in hardware. The lack of a dense electronic element makes it difficult to construct such massive amounts of dense electronic systems. However, memristor structures with resistive switching characteristics offer significant advantages for performing certain synaptic functions and for the construction of dense electronic systems. Memristor structures, which are highly similar to neural synapses with permanent memory and nonlinear conductance changes, have the superior advantage of performing magnificent synaptic functions such as short/long-term-plasticity (STP/LTP), paired-pulse facilitation (PPF), and spike-time-dependent-plasticity (STDP).^15−19^ Studies have shown that memristor structures can be produced using various oxide materials such as SiO_2_, TaO, VO_2_, ZnO, CuO, NiO, and TiO_2._^20^ Among these compounds, the Mott insulator VO_2_ recently has been intensively studied due to the large conductance changes at room temperature. Recent researches indicate that the resistive switching behavior is caused by nanosized conductive filament structures resulting from the mobile oxygen vacancies in the VO_2_ structure.^21,22^

Recently, memristors based on Mott insulators such as VO_2_ have been studied intensively due to the metal–insulator transition being just above room temperature. This reversible phase transition can be controlled by electrical pulses. The response change of its measurable electrical conductivity resulting from phase change and oxygen vacancies in its structure makes VO_2_ one of the most important candidates in fields such as memory devices, processors, and sensor technologies. The increment in the resistivity dissipates itself with time and is reconstructed with new electrical pulses, similar to the forgetting and learning mechanism in biological neurons. Zhou et al., show that VO_2_-based switching devices are ultrafast and can reach a reliability level of 2 times the magnitude of the ON/OFF ratio in a period of 2 ns.^23,24^ Therefore, VO_2_ can be used in many memory devices, including resistive random-access memory (ReRAM), 3D memory array and independent VO_2_/TiO_2_ console.^25,26^ In addition, VO_*x*_-based memristor devices provide high reliability, efficiency, and low power consumption.^27,28^ In most published articles, VO_2_-based, artificial synaptic devices were grown on a rigid substrate. Zhenfa Wu et al. reported a flexible synaptic transistor on a polyimide substrate is designed and fabricated using a solid-state electrolyte gate and a VO_2_ Mott insulator thin film.^29^ Therefore, VO_2_ has the potential for wearable electronics. There is great interest in VO_*x*_-based memristive devices, especially in the construction of matrix multiplication or cross-bar-arrays to be used in performing high data-intensive tasks such as artificial neural networks.^30,31^ By using the electron beam lithography technique, it is possible to fabricate a high-density 3D cross-bar array for the hardware implementation of neuromorphic computing.^32^ Additionally, 2D metal oxides and porous crystalline materials are among the structures that have recently attracted great attention from researchers working on memory and neuromorphic computing systems.^33,34^

In this study, we report on the fabrication and structural characterization of the VO_*x*_-based memristor devices which are grown by using a VO_2_ PLD (Pulsed Laser Deposition) target. The resistive switching behavior and synaptic functions such as LTD, LTP, PPF, and STDP have been successfully observed in fabricated memristor devices.

## 2 Experimental Details

**Figure 1:**
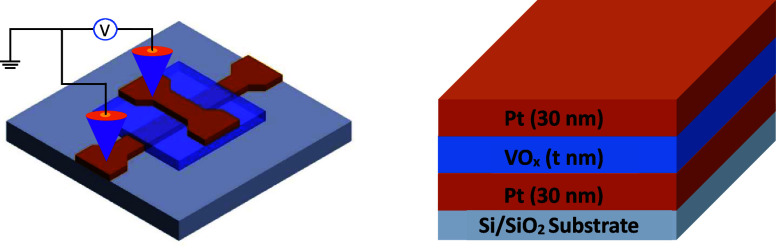
Schematic picture of the single-cell memristor devices, measurement setup, and stack figure of the device.

In this study, memristor devices in the stack structure of Si/SiO_2_/Pt/VO_*x*_/Pt have been grown by using Pulsed Laser Deposition (PLD) and DC Magnetron Sputtering in a base pressure of 10^–9^ mbar. After chemical and thermal cleaning of the commercially available self-oxidizing Si(100) substrate, 30 nm thick sub-Pt electrodes were grown on the substrates in a DC magnetron sputtering chamber. Then they were transferred to the PLD chamber and VO_*x*_ thin film structures of 25, 13, and 3 nm thickness were deposited on the bottom electrodes using the pulsed laser deposition (PLD) method. These two growth chambers are connected via a connection system, in which vacuum conditions are maintained. The distance between the VO_2_ PLD target and substrates was kept constant at 24.8 mm during the growth of VO_*x*_ oxide layers in the PLD system. In this system, a KrF excimer laser (λ = 248 nm) was operated at 350 mJ and had a repetition rate of 10 Hz. The laser fluence was set to 2.4 J/cm^2^. All PLD parameters remained the same throughout the growth of oxide layers, and all substrates were spun at a low speed during deposition. Finally, the 30 nm Pt top electrode has been sputtered. A metallic mask was used to grow the contacts that will provide an electrical connection through the lower and upper electrodes, as shown schematically in Figure 1. The surface area of the top electrode in the device region is about 1 mm^2^.

In electrical characterizations, the bottom electrodes were always grounded, and DC voltages or AC pulse voltages were applied to the upper electrodes. All electrical measurements were conducted under atmospheric conditions at room temperature. The Keithley 2450 source meter and Tektronix AFG 3100 series arbitrary function generator were utilized for DC and AC electrical characterizations. Electrical measurements were carried out with measurement setups created by using the LabVIEW software interface. X-ray photoelectron spectroscopy (XPS) technique was used to determine the chemical composition and oxidation state of the VO_*x*_ active structure. Additionally, the Photoluminescence (PL) Spectroscopy technique was used to confirm the presence of oxygen vacancy defects in the VO_*x*_ active structure. The thickness of the thin films was determined by using the surface profilometer technique.

## 3 Result and Discussion

**Figure 2:**
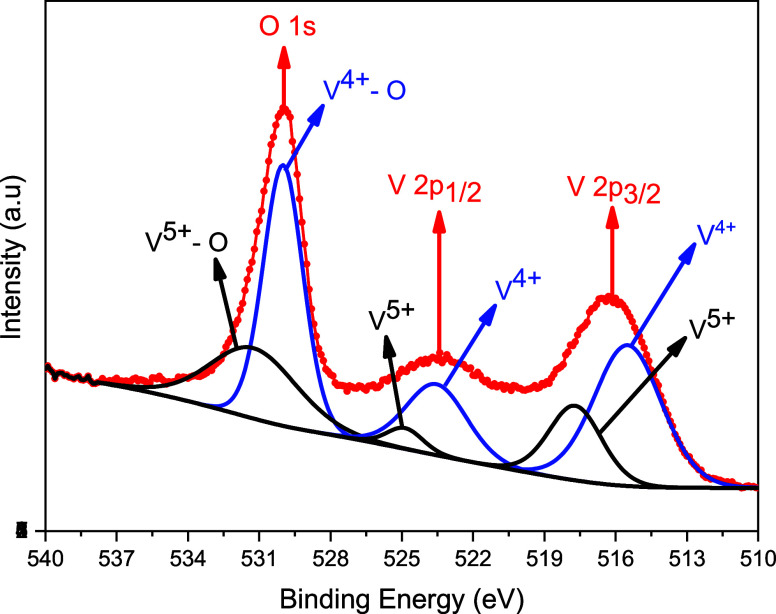
Experimental high-resolution XPS Spectrum of O 1s and V 2p environment and the simulation results of experimental data. The red solid circle shows experimental data, and the red solid line shows a fitting envelope. CPS refers to the counts per second.

To examine the composition rates of the surface of VO_*x*_ film and oxidation states of Vanadium element, X-ray photoelectron spectroscopy (XPS) measurements were performed on as-grown VO_*x*_ thin films. Figure 2 shows the high-resolution XPS spectra for VO_*x*_ thin film in the binding energy range of 540 and 510 eV at room temperature and the simulation of experimental XPS spectra by using CasaXPS software.^35^ In this energy range, the peaks originating from the oxygen (left-hand side peak in Figure 2) and vanadium (right-hand side peaks in Figure 2) ions are observed together. V peaks show a typical two peaks (2p_1/2_ and 2p_3/2_) structure due to the spin–orbit coupling. The binding energies for these V 2p_3/2_ and V 2p_1/2_ peaks correspond to values of 516.3 and 523.4 eV, respectively. During the fitting process, the area ratio between V 2p_3/2_ and V 2p_1/2_ is fixed at 2:1. It is seen from Figure 2, that the peaks belonging to V^4+^ are broadened due to the contribution of the higher valence state (5+) of the V ion. The relative peak intensity of V^4+^ in the spectrum is larger than the intensity of the peaks corresponding to V^5+^. High-intensity peaks (V^4+^) have been attributed to the stoichiometric VO_2_ and low-intensity peaks have been attributed to the V_2_O_5_ phases of VO_2_. Clearly, the binding energies obtained from the fitting of experimental data for the 2p_3/2_ and 2p_1/2_ of V^4+^ are 515.8 and 523.3 eV (splitting is 7.5 eV) respectively. Similarly, the binding energies obtained from the fitting of experimental data for the 2p_3/2_ and 2p_1/2_ of V^5+^ are 517.8 and 524.9 eV (splitting is 7.1 eV) respectively. The obtained fit values of binding energy are in good agreement with the literature.^36^ The V^5+^ peak here arises only from oxidation of the film surface. However, its relatively low content compared to V^4+^ confirms that the oxygen in the VO_2_ structure is not only due to the oxidation of the film surface, but is primarily due to the strong bonding of Vanadium with oxygen.^37,38^ No significant change in the spectrum and binding energies was observed with varying film thicknesses.

**Figure 3:**
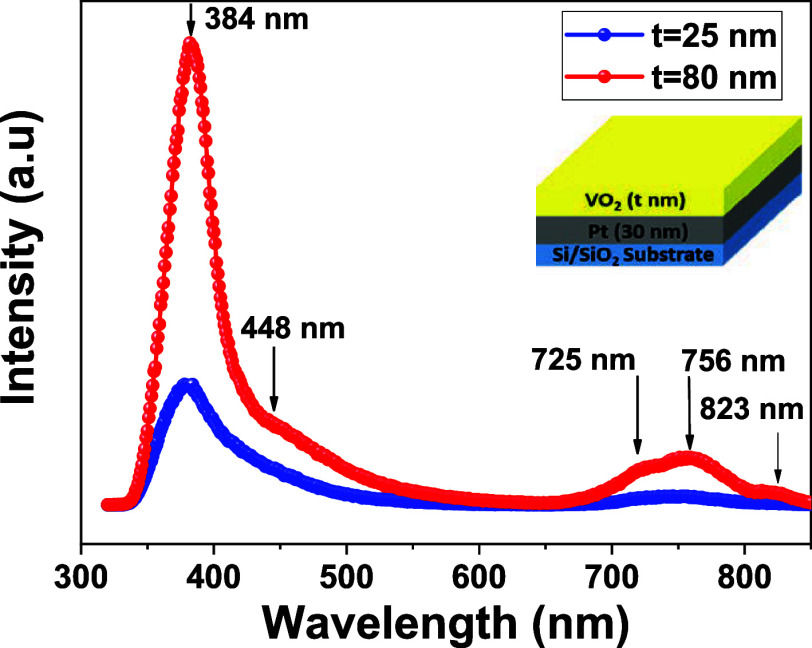
Room temperature Photoluminescence (PL) spectra of VO*_x_* thin films with increasing film thickness in the order. The inset shows the stack of the measured film with different thicknesses.

The PL method is a common technique used to characterize the nature of the intrinsic defects such as oxygen vacancies in various systems.^39^ Because the oxygen vacancies have a significant effect on changing the conductivity with the applied electric field in metal oxides. Therefore, PL measurements were performed on VO_*x*_ thin films to ensure the presence of oxygen vacancies in the fabricated VO_*x*_-based memristor devices. Figure 3 shows the results of Photoluminescence (PL) measurements performed at room temperature for VO_*x*_ thin films of different thicknesses on Pt/SiO_2_(substrate). All samples were excited with an Agilent Cary Eclipse fluorescence spectrophotometer with a 275 nm wavelength laser beam. Although PL measurements are rare in literature for the VO_2_ thin films on different substrates, similar PL spectra have been observed in Reff.^40−42^ As seen in Figure 3, there are five emission peaks centered at 384–448, 725, 756, and 823 nm, respectively. The most intensive excitation peak at 384 nm can be assigned to the free-excitation emission.^41,43−45^ The excitation peaks around ∼448 nm may be attributed to the electric charge transfer corresponding to the bonding energy of O=V and O–V–O bonds.^46^ The emission peaks around 750 nm are related to the transmission of d electrons of Vanadium to the p orbitals holes of Oxygen or may be due to the oxygen vacancies in VO_*x*_ thin film structures.^21,44,47^ While the excitation peaks occurred at a narrower wavelength with increasing film thickness, the emission peaks arising from the oxygen vacancy became more prominent with increasing film thickness. As a result, the PL spectrum clearly revealed that oxygen vacancies exist in VO_*x*_ thin films and these oxygen vacancies tend to increase with increasing film thickness.

**Figure 4:**
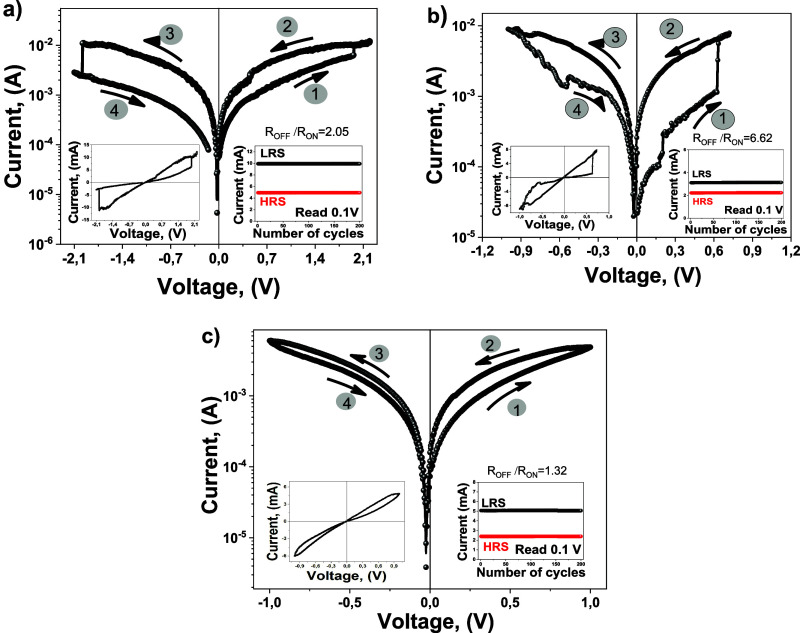
Current voltage characteristics for VO_*x*_-based memristor devices with different thicknesses. (a) for the VO_*x*_ layer of 25 nm thickness, (b) for the VO_*x*_ layer of 13 nm thickness (c) for the VO_*x*_ layer of 3 nm thickness. Left left-handed inset shows the linear scale *I*–*V* curves, and the right-handed inset shows the endurance characteristics of each memristitor device.

Figure 4 shows the characteristic current–voltage measurements and endurance characteristics of VO_*x*_-based single-cell memristor devices with different oxide thicknesses. The current–voltage graphs obtained for all devices exhibited a pinched hysteresis loop, a defining characteristic of memristor devices. When positive voltage was applied to the upper electrodes of the devices, a gradual increase in currents occurred, causing a transition from a high resistance state (HRS) to a low resistance state (LRS) in the devices, known as the SET process. These changes indicate that the devices were initially in high resistance states. Conversely, when negative voltage was applied to the upper electrodes, a gradual decrease in the currents occurred, and the reset process took place, which is the opposite of the set process. In other words, the resistance of the devices changed from low resistance to high resistance. According to the current–voltage characteristics obtained for all devices, the conductivity of the devices could be changed by applying appropriate positive and negative voltages, and it is observed that all devices have bipolar resistance switching characteristics. Some differences were observed in the set and reset voltages of devices consisting of VO_*x*_ structures with different thicknesses. The characteristic SET and RESET voltages were determined by the maximum of the derivative of the *I*–*V* curve. The magnitude of the set and reset voltages for the sample with the 25 nm VO_*x*_ layer is about ±2 V. The current–voltage behavior of the 3 nm VO_*x*_ layer sample shows a gradual process. Even if it is gradual, the maximum of the derivative of the *I*–*V* curve shows the magnitude of the set and reset voltages is about ±0.94 V. On the other hand, due to the sharp changes in the *I*–*V* curve for the sample of 13 nm oxide thickness the set voltage is 0.64 V, and the reset voltage is about 0.85 V. Similar to the set and reset voltages, differences were observed in the maximum current values of devices with different VO_*x*_ thicknesses. While the maximum current value that the device with a thickness of 25 nm could reach is 9.6 mA, this value was approximately 6.6 mA for the 13 nm thick device, and it was 4.2 mA for the 3 nm thick device. These values obtained according to the thickness function show that the film thickness is the determining factor in the maximum current values and set and reset voltage values of the device. The individual maximum currents of all devices were almost equal during positive and negative scanning operations. Both this situation and the gradual change of the currents reveal that the switching is predominantly caused by the conductive filament structures formed by the oxygen vacancies in the VO_*x*_ structure.^48^ As a result, the obtained switching characteristics revealed that the produced structures have memristive properties.

One of the other important parameters of the memristor devices is the *R*_OFF_/*R*_ON_ ratio, which is large for a larger hysteresis loop area. For the sample of 25 nm VO_*x*_ layer, *R*_OFF_/*R*_ON_ = 2.05 is estimated for the applied voltage of 1.675 V, for the sample of 13 nm VO_*x*_ layer, *R*_OFF_/*R*_ON_ = 6.62 is estimated for the applied voltage of 0.525 V, and for the sample of 3 nm VO_*x*_ layer, *R*_OFF_/*R*_ON_ = 1.32 is estimated for the applied voltage of 0.835 V. The right insets of Figure 4 show the endurance behavior of VO_*x*_-based memristor devices with different thicknesses. The corresponding current values for the high resistance state (HRS) and low resistance state (LRS) of the devices were examined with the corresponding SET and RESET voltage values in their current–voltage characteristics. The endurance behavior obtained for memristor devices clearly shows that these devices can operate very stably between LRS and HRS. Additionally, it was observed that with decreasing VO_*x*_ thickness, the corresponding currents for LRS and HRS tended to decrease according to the 25 nm thick device. So it is attributed that with decreasing VO_*x*_ film thickness, the resistance of the devices increases and the amount of oxygen vacancy in the VO_*x*_ structures decreases.

**Figure 5:**
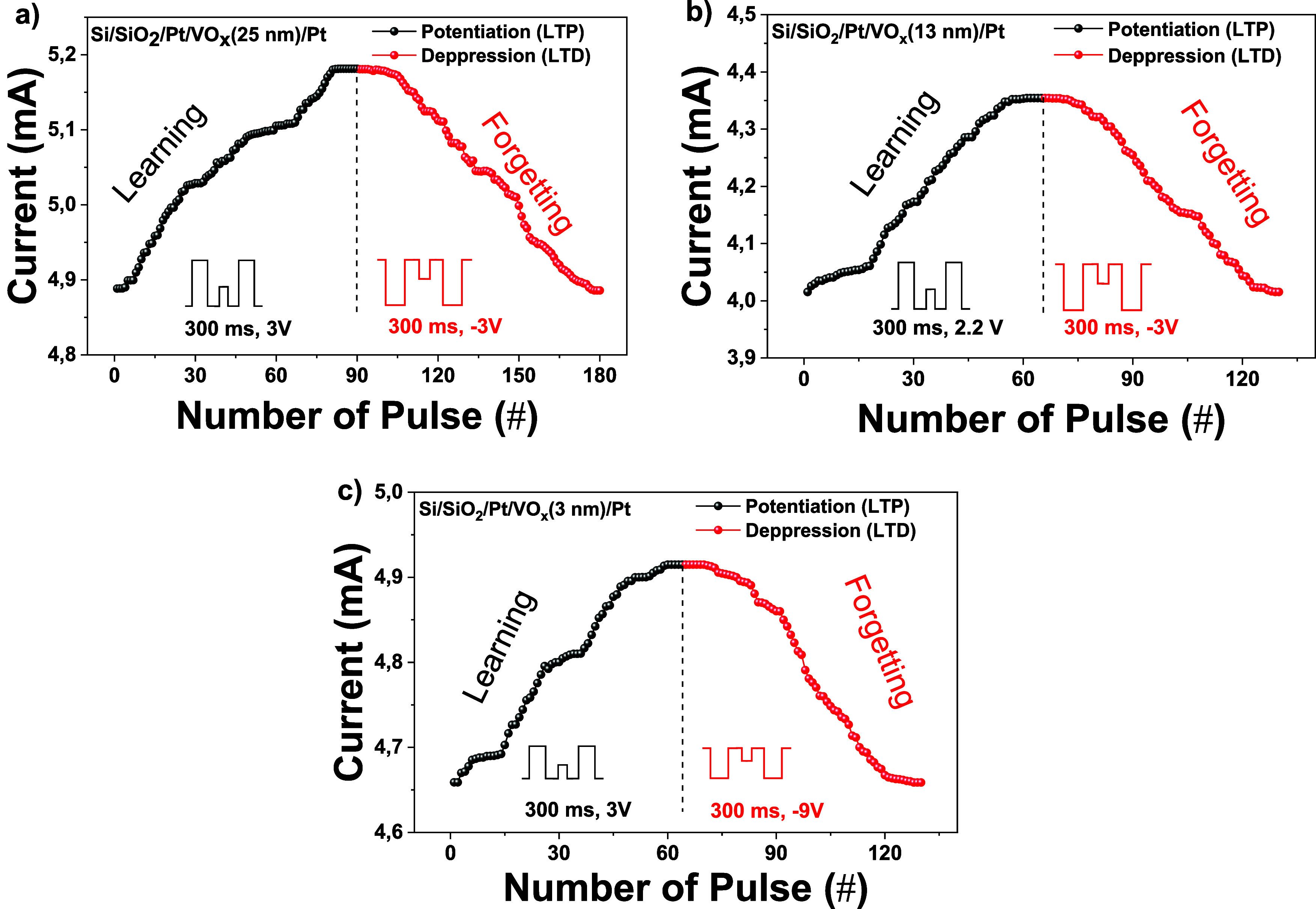
Gradual current change of the memristor devices against applied consecutive positive and negative pulses. (a) Memristor device with 25 nm VO_*x*_ thickness, (b) 13 nm VO_*x*_ thickness, and (c) 3 nm VO_*x*_ thickness.

The characteristic synaptic plasticity observed in neuromorphic systems is the key to learning and memory abilities. Remarkably, potentiation and depression capabilities at biological synapses are often achieved through successive excitatory spikes.^8,49^ Making an analogy to this mechanism in biological systems, we tried to obtain potentiation and depression structures similar to those in biological systems by applying consecutive positive and negative pulses to our memristor devices. Figure 5 shows gradual current changes in memristor devices with the application of successive positive and negative pulses. These gradual current changes occurred in the form of potentiation and depression of the biological synapses. As seen in Figure 5, potentiation responses were obtained by applying 90 positive pulses in the form of 3 V amplitude and 300 ms pulse-width to the device consisting of a 25 nm thick VO_*x*_ film. Similarly, depression responses were obtained by applying 90 negative pulses in the form of a 3 V amplitude and 300 ms pulse-width to the device consisting of a 25 nm thick VO_*x*_ film. To obtain the potentiation response for the device consisting of a 13 nm thick VO_*x*_ layer, successive pulses of 2.2 V amplitude with the same pulse width were applied. To obtain a depression response in this device, pulses of 300 ms width and 3 V amplitude were applied. For the device with a 3 nm thickness VO_*x*_ layer, by applying 3 V 300 ms pulses, the potentiation response was obtained, and by applying 9 V 300 ms pulses, the depression response was obtained. In addition, the intervals of all consecutive pulses were 5 ms, and the current changes after the pulses were measured by a 0.1 V reading voltage. In our measurements, we had to use pulses of different amplitudes to switch from the potentiation response at the saturation point to the depression response, similar to that observed in biological synapses. It is attributed entirely to the film thickness. Additionally, the current changes and current saturation values of each device are different from each other in the gradual current responses obtained, and the number of pulses is different for each device to achieve full reinforcement and depression responses. For example, more stimulating pulses were suitable for a device with 25 nm oxide thickness. For all of these differences, it is attributed that the thickness of the oxide layer in the devices is an important factor affecting synaptic properties. From all these gradual current responses, we can say that the gradual current changes that occur as a result of successive pulses called potentiation and depression correspond to the LTP and LTD mechanisms observed in artificial synapses.^49−52^ Normally it is not always possible to obtain an equivalent LTP behavior after LTP with the same voltage amplitudes, and the current change cannot be achieved at the desired level. As examples can be seen in the literature, appropriate LTD mechanisms were obtained by applying amplitudes with different voltage values.^15,53^ This situation was achieved by trying many voltage values to obtain the most suitable LTD mechanism.

**Figure 6:**
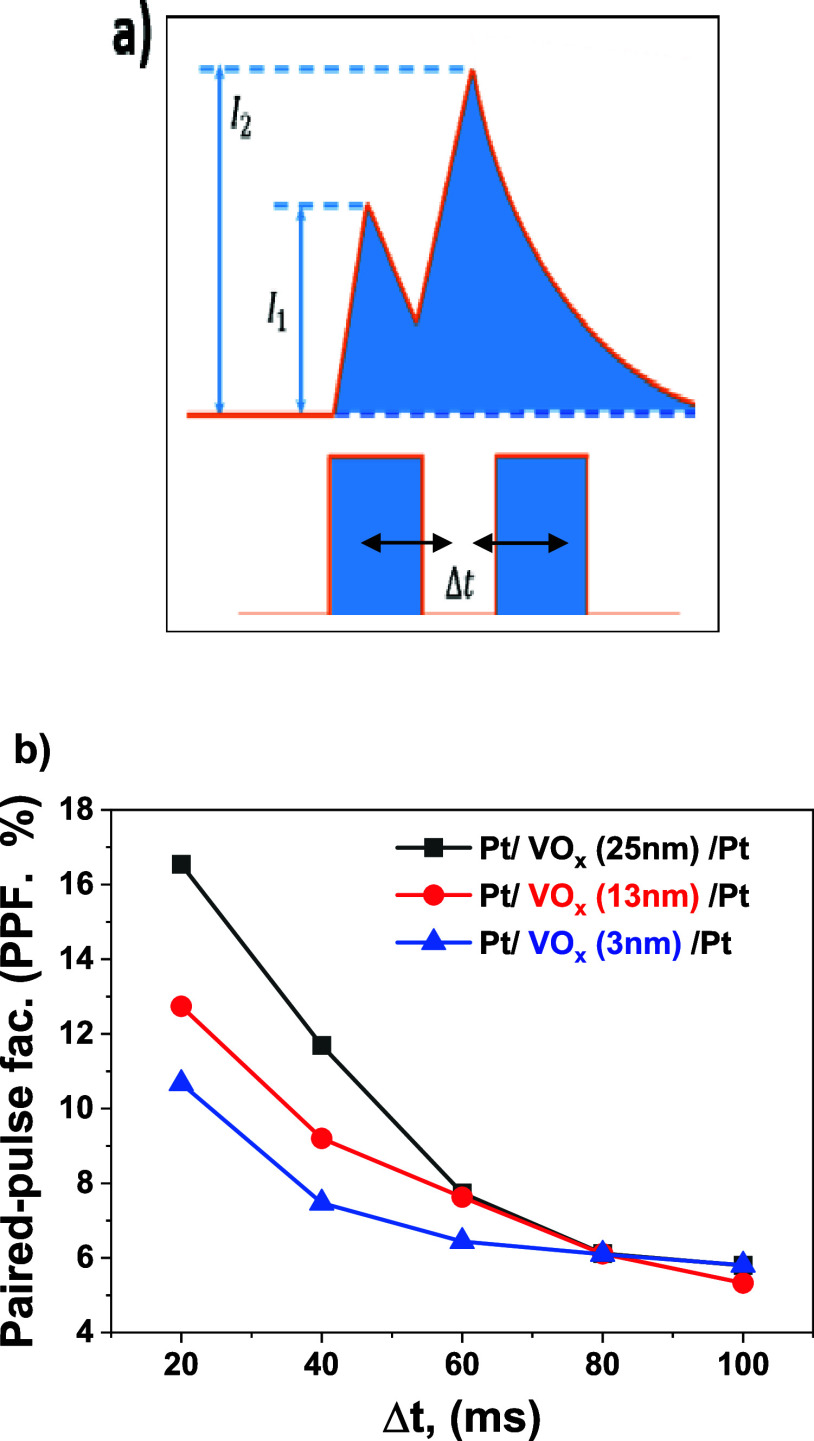
Dynamics applied for PPF indices and Paired pulse facilitation (PPF) indices for devices. (a) Obtaining the PPF mechanism and (b) PPF indices of memristor devices with 25, 13, and 3 nm VO_*x*_ thickness.

Figure 6 shows the current changes that occurred when two successive voltage pulses were applied to VO_*x*_-based single-cell memristor devices. The current changes observed with the effect of these applied pulses resemble the paid-pulse facilitation (PPF) relationship in biological synapses. Studies have shown that memristor devices can perform important neural tasks by stimulating them with appropriate pulse pairs and changing their conductivity.^11,15,16,19^ Among these tasks, the PPF function is an important short-term logic function. The PPF mechanism is achieved in biological systems by the sequential application of two presynaptic spikes. In this case, the second applied spike produces a more notable response than the first.^16,54^ Inspired by the PPF mechanism in biological synapses, we aimed to demonstrate the PPF function in our memristor devices by applying consecutive pulse pairs with different pulse intervals. Here, we changed the time interval between pulse pairs by giving different values, especially to establish complete similarity with the structures in biological systems. The purpose of proceeding in this way was to examine deeply the effect of the time interval between pulse pairs on current changes. The PPF characteristic in memristor devices is determined using a special equation as given below;
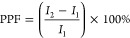
here, *I*_1_ and *I*_2_ are the current responses created by the first and second pulses, respectively.^55^ As seen in Figure 6b, changes in the PPF function are observed as a function of the interval between the pulse pairs. Here the currents were measured after the first and second pulses were applied. For each device, pulses with 2 V pulse amplitude and 300 ms pulse width were applied with different pulse intervals. Although the percentage PPF values obtained are some low, generally a smaller pulse interval resulted in a larger PPF index. And with increasing time intervals, PPF function has tended to decrease. However, depending on the function of the time interval, the behavior of the devices and their saturation points were quite different. Especially with the decrease in the thickness of the oxide layer, significant decreases were observed in the first time interval values of PPF. In general, lower PPF values were obtained as the film thickness decreased. It is attributed that the decrease in oxygen vacancies due to the decrease in film thickness was effective in the decrease in PPF. Still, the PPF values obtained for all devices were always positive. This situation shows us that the current values obtained as a result of the second pulse stimulation were always greater than the current values obtained as a result of the first applied pulse. For this observed behavior, we can draw a similarity between the memristor devices we produced and the PPF behavior in biological synapses. The results obtained have clearly demonstrated that there is a deep relationship between the conductivity changes of the devices and the time interval between pulse pairs. This relationship provides a significant advantage. If you do not have a strong input stimulation, you can make a significant change in the conductivity of the device by using pulse stimulation with very narrow time intervals.^16^ In addition, when we examine the studies for PPF behavior in the literature in detail, we can say that the model suitable for the PPF behavior obtained in this study is the conductive filament model.^56,57^ Because when the current responses were examined, the effect of the second pulse stimulation was always greater than the effect of the first pulse stimulation. The factor that produces this effect is the oxygen vacancies within the VO_*x*_ structure. Based on the conductive filament model mentioned above, the mechanism of PPF can be explained as follows. The oxygen vacancies that move with the application of the first pulse stimulation form conductive filaments. Then, with a second pulse stimulation, the number of these conductive filament structures increases, resulting in a further increase in conductivity. However, for the PPF functions obtained at low rates, it is understood that conductive filament structures are not formed in sufficient numbers with these pulses. Using the conductive filament model, we can also explain the decreasing PPF function with an increasing pulse interval. Because, with an increasing pulse interval, the conductive filament structures formed in the initial state may deteriorate and return to their previous state. Thus, there is a decrease in device conductivity compared with the lower pulse interval.

A normal biological synapse structure consists of the connection of a presynaptic neuron and a postsynaptic neuron. This connection is achieved through the synaptic cleft. If we draw an analogy with biological synaptic structures, in memristor devices that serve as artificial synapses, the upper and lower contacts serve as neurons. The oxide layer, which plays a key role in switching in these devices, provides the synaptic structure. In addition, with the voltage stimulation applied to these devices, increases and decreases in the conductivity of the device can be achieved, and similarities can be established with the strengthening and collapse dynamics in biological systems. Moreover, the electrical conductivity of the device has the potential to compensate for synaptic weight changes in biological systems.^58^ STDP, which occurs from the temporal arrangement of previous and subsequent synaptic spikes, is a weight change and is one of the important learning rules. Especially STDP has a high potential to achieve learning ability in hardware-based spiking neural networks.^59^

**Figure 7:**
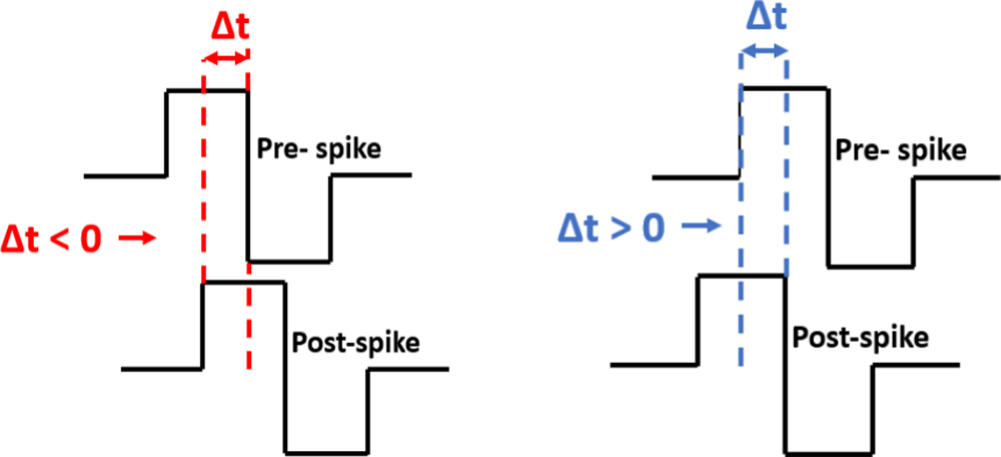
Implemented dynamics for the STDP function.

**Figure 8:**
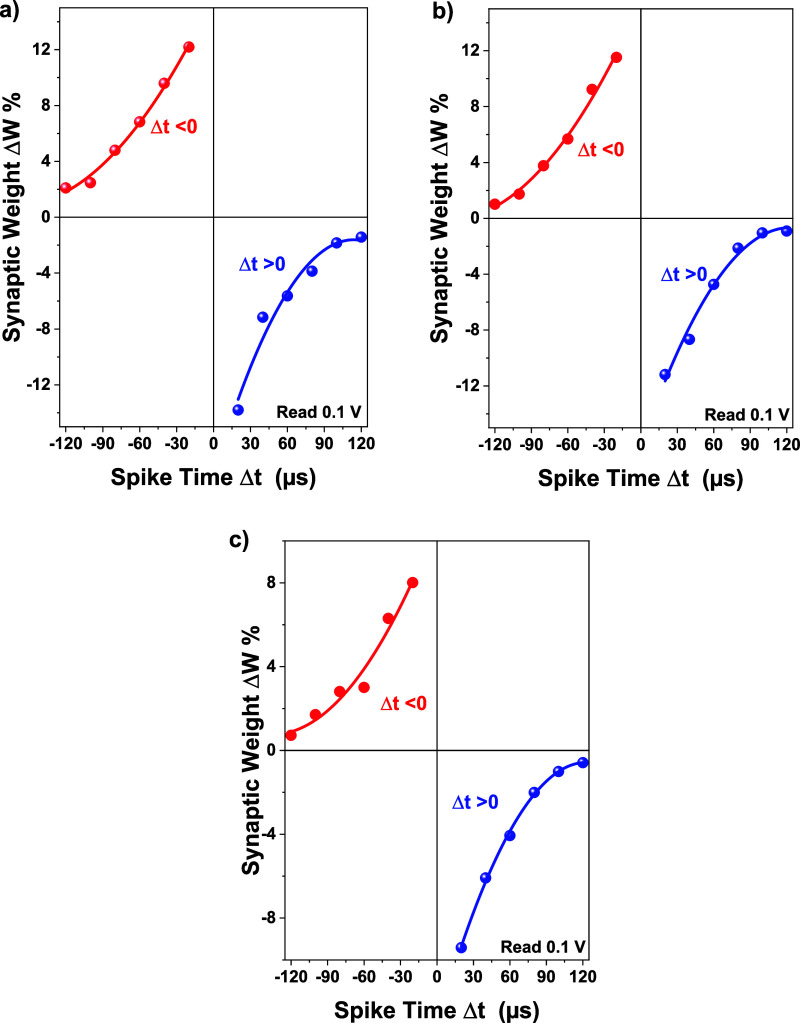
STDP curves for VO_*x*_-based memristor devices with different thicknesses. (a) Memristor device with 25 nm VO_*x*_ thickness, (b) 13 nm VO_*x*_ thickness, (c) 3 nm VO_*x*_ thickness.

In this study, we applied the usual STDP mechanism shown in Figure 7 to our memristor-based artificial synaptic devices. If the prespike pulse occurs before the postspike pulse. The current response of the device increases and Δ*t* < 0. Such a situation represents a strengthening of the connection strength between two neurons in biological systems. Conversely, if the prespike pulse lags behind the postspike pulse. The current response of the device decreases and Δ*t* > 0. This indicates a weakening of the connection strength between two neurons in biological systems.^60^ The relative time arrangement of prespike and postspike in these two processes determines the nature of the synaptic weight change. If we represent this synaptic weight change with a mathematical expression in percentage value, we can use an equation such as the one below.
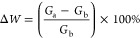
Here, *G*_b_ is the conductance measured before stimulation and *G*_a_ is the conductance measured after stimulation of prespike and postspike pairs.^19,61^ In this study, we wanted to establish analogies with the STDP rule in biological systems by applying pulse pairs to VO_*x*_-based memristor devices. For our memristor devices, we applied these pulse pairs of 2 and −2 V pulses with 120 μs pulse width corresponding to prespike and postspike structures, respectively. Figure 8 shows the synaptic weight changes in the memristor devices with different thicknesses. As seen in the figures, there is a strong relationship between Δ*W* and Δ*t*. For all devices, when Δ*t* > 0, an increase in Δ*W* value was observed with decreasing Δ*t*. This increase in Δ*W* value naturally indicates that the connection strength between two neurons decreases. However, the maximum Δ*W* values of the devices differed depending on the thickness of the oxide layer. When Δ*t* > 0, the maximum Δ*W* value was reached in the 25 nm thick VO_*x*_-based device. It is attributed that the thickness of the oxide layer that functions as the synaptic structure is effective in this case. We can talk about a similar relationship between Δ*W* and Δ*t* when Δ*t* < 0. In this case, this increase in Δ*W* value shows that, unlike the first case, the connection strength between the two neurons has increased. The maximum Δ*W* value was observed in the device with a 25 nm thick oxide layer, as in the first case. However, the Δ*W* values in the device with a 3 nm thick oxide layer were relatively lower than the other two devices. The synaptic weight changes we observed according to the relative change of oxide film thickness in the devices showed that the thickness of the oxide layer, and especially the amount of oxygen vacancy that varies depending on the thickness of the oxide layer have an important effect on the synaptic performance of the devices. As a result, based on the synaptic weight changes, it is observed that the VO_*x*_-based memristor devices produced in this study have the potential to be used as artificial synapses.

## 4 Conclusions

XPS results confirmed that the oxide layers of the memristor devices were formed in the form of VO_*x*_ with a good stoichiometric ratio. No change in the VO_*x*_ form and stoichiometric ratio was observed with increasing oxide layer thickness. The results of the PL spectrum clearly showed that there are oxygen vacancies within the VO_*x*_ structure, which is the oxide layer, and the amount of oxygen vacancies tends to increase with increasing film thickness. From the *I*–*V* graphs, we observed that all of the devices had a pinched hysteresis curve, which is considered a fingerprint for memristor devices. Electrical behavior has shown that memristor devices have bipolar resistive switching behavior. The set and reset values of devices with different thicknesses of oxide layers were different. By applying sequential pulses in all devices, gradual current changes resembling the LTP and LTD mechanisms observed in biological synapses were obtained. In these measurements, it was understood that the thickness of the oxide layer in the devices is an important factor that can affect the LTP and LTD parameters. Although PPF values for all devices were low, larger PPF indices were obtained at smaller pulse intervals, which is quite similar to biological systems. In particular, the synaptic weight changes obtained showed that these VO_*x*_-based memristor devices can be used as artificial synapses. Energy consumption in a single memristor per synaptic event depends on the amplitude of the applied voltage pulse, the pulse with it, and the response current. For our devices, these values are 3 V, 0.3 mA, and 300 ms, respectively. Thus, the average energy consumption per synaptic event for our devices was estimated to be 270 μJ. Recently different types of memristor devices with very low energy consumption have been reported. For our memristor devices, the surface area of the top electrode is about 1 mm^2^. Therefore, the estimated high energy consumption is a result of the measured high current during one synaptic event.

There are many factors that affect the performance of memristors. One of these factors is the chemical composition of the switching layer. For instance, in VCM memristors, the memristive effects are driven by the oxygen vacancy. Also, the epitaxial growth of the metal-oxide film, roughness between interfaces, the surface area of the devices, and the general morphology of the memristor affect the performance of the memristor devices.

## Data Availability

The data underlying this study are not publicly available due to no suitable repository exists for hosting data in this field of study. The data are available from the corresponding author upon reasonable request [list any registration or other requirements for access].

## Accession Codes

All data produced by authors are presented in this manuscript and they are available in DOI.

## References

[ref1] Zhuge X.; Wang J.; Zhuge F. Photonic Synapses for Ultrahigh-Speed Neuromorphic Computing. Phys. Status Solidi RRL 2019, 13 (9), 1900082 10.1002/pssr.201900082.

[ref2] Wang J.; Zhuge F. Memristive Synapses for Brain-Inspired Computing. *Adv*. Mater. Technol. 2019, 4 (3), 1800544 10.1002/admt.201800544.

[ref3] Lv Z.; Wang Y.; Chen J.; Wang J.; Zhou Y.; Han S.-T. Semiconductor Quantum Dots for Memories and Neuromorphic Computing Systems. Chem. Rev. 2020, 120 (9), 3941–4006. 10.1021/acs.chemrev.9b00730. 32202419

[ref4] Zhu J.; Zhang T.; Yang Y.; Huang R. A Comprehensive Review on Emerging Artificial Neuromorphic Devices. *Appl*. Phys. Rev. 2020, 7 (1), 011312 10.1063/1.5118217.

[ref5] Tang J.; Yuan F.; Shen X.; Wang Z.; Rao M.; He Y.; Sun Y.; Li X.; Zhang W.; Li Y.; Gao B.; Qian H.; Bi G.; Song S.; Yang J. J.; Wu H. Bridging Biological and Artificial Neural Networks with Emerging Neuromorphic Devices: Fundamentals, Progress, and Challenges. Adv. Mater. 2019, 31 (49), 1902761 10.1002/adma.201902761. 31550405

[ref6] Xia Q.; Yang J. J. Memristive Crossbar Arrays for Brain-Inspired Computing. Nat. Mater. 2019, 18 (4), 309–323. 10.1038/s41563-019-0291-x. 30894760

[ref7] Ielmini D. Brain-Inspired Computing with Resistive Switching Memory (RRAM): Devices. Synapses and Neural Networks. Microelectron Eng. 2018, 190, 44–53. 10.1016/j.mee.2018.01.009.

[ref8] Hoffmann A.; Ramanathan S.; Grollier J.; Kent A. D.; Rozenberg M. J.; Schuller I. K.; Shpyrko O. G.; Dynes R. C.; Fainman Y.; Frano A.; Fullerton E. E.; Galli G.; Lomakin V.; Ong S. P.; Petford-Long A. K.; Schuller J. A.; Stiles M. D.; Takamura Y.; Zhu Y. Quantum Materials for Energy-Efficient Neuromorphic Computing: Opportunities and Challenges. APL Mater. 2022, 10 (7), 070904 10.1063/5.0094205.

[ref9] Kumar S.; Wang X.; Strachan J. P.; Yang Y.; Lu W. D. Dynamical Memristors for Higher-Complexity Neuromorphic Computing. Nature Reviews Materials. 2022, 7, 575 10.1038/s41578-022-00434-z.

[ref10] del Valle J.; Ramírez J. G.; Rozenberg M. J.; Schuller I. K. Challenges in Materials and Devices for Resistive-Switching-Based Neuromorphic Computing. J. Appl. Phys. 2018, 124 (21), 211101 10.1063/1.5047800.

[ref11] Jo S. H.; Chang T.; Ebong I.; Bhadviya B. B.; Mazumder P.; Lu W. Nanoscale Memristor Device as Synapse in Neuromorphic Systems. Nano Lett. 2010, 10 (4), 1297–1301. 10.1021/nl904092h. 20192230

[ref12] Yang J. J.; Strukov D. B.; Stewart D. R. Memristive Devices for Computing. Nat. Nanotechnol 2013, 8 (1), 13–24. 10.1038/nnano.2012.240. 23269430

[ref13] Linn E.; Rosezin R.; Kügeler C.; Waser R. Complementary Resistive Switches for Passive Nanocrossbar Memories. Nat. Mater. 2010, 9 (5), 403–406. 10.1038/nmat2748. 20400954

[ref14] Rahimi Azghadi M.; Chen Y.-C.; Eshraghian J. K.; Chen J.; Lin C.-Y.; Amirsoleimani A.; Mehonic A.; Kenyon A. J.; Fowler B.; Lee J. C.; Chang Y.-F. Complementary Metal-Oxide Semiconductor and Memristive Hardware for Neuromorphic Computing. Advanced Intelligent Systems 2020, 2 (5), 1900189 10.1002/aisy.201900189.

[ref15] Liu C.; Cao Y.-Q.; Wu D.; Li A.-D. Simulation of Biologic Synapse Through Organic-Inorganic Hybrid Memristors Using Novel Ti-Based Maleic Acid/TiO _2_ Ultrathin Films. IEEE Electron Device Lett. 2020, 41 (1), 155–158. 10.1109/LED.2019.2956282.

[ref16] Xu Z.; Li F.; Wu C.; Ma F.; Zheng Y.; Yang K.; Chen W.; Hu H.; Guo T.; Kim T. W. Ultrathin Electronic Synapse Having High Temporal/Spatial Uniformity and an Al2O3/Graphene Quantum Dots/Al2O3 Sandwich Structure for Neuromorphic Computing. NPG Asia Mater. 2019, 11 (1), 18 10.1038/s41427-019-0118-x.

[ref17] Li Y.; Ang K.-W. Hardware Implementation of Neuromorphic Computing Using Large-Scale Memristor Crossbar Arrays. Advanced Intelligent Systems 2021, 3 (1), 2000137 10.1002/aisy.202000137.

[ref18] Kim S. J.; Kim S.; Jang H. W. Competing Memristors for Brain-Inspired Computing. iScience 2021, 24 (1), 101889 10.1016/j.isci.2020.101889. 33458606 PMC7797931

[ref19] Ilyas N.; Li D.; Li C.; Jiang X.; Jiang Y.; Li W. Analog Switching and Artificial Synaptic Behavior of Ag/SiOx:Ag/TiOx/P++-Si Memristor Device. Nanoscale Res. Lett. 2020, 15 (1), 30 10.1186/s11671-020-3249-7. 32006131 PMC6994582

[ref20] Hu L.; Han W.; Wang H. Resistive Switching and Synaptic Learning Performance of a TiO _2_ Thin Film Based Device Prepared by Sol–Gel and Spin Coating Techniques. Nanotechnology 2020, 31 (15), 155202 10.1088/1361-6528/ab6472. 31860903

[ref21] Novodvorsky O. A.; Parshina L. S.; Lotin A. A.; Mikhalevsky V. A.; Khramova O. D.; Cherebylo E. A.; Panchenko V. Ya. Vanadium- and Titanium Dioxide-Based Memristors Fabricated via Pulsed Laser Deposition. *Journal of Surface Investigation: X-ray, Synchrotron and Neutron*. Techniques 2018, 12 (2), 322–327. 10.1134/S1027451018020313.

[ref22] Yi W.; Tsang K. K.; Lam S. K.; Bai X.; Crowell J. A.; Flores E. A. Biological Plausibility and Stochasticity in Scalable VO2 Active Memristor Neurons. Nat. Commun. 2018, 9 (1), 4661 10.1038/s41467-018-07052-w. 30405124 PMC6220189

[ref23] Zhou Y.; Chen X.; Ko C.; Yang Z.; Mouli C.; Ramanathan S. Voltage-Triggered Ultrafast Phase Transition in Vanadium Dioxide Switches. IEEE Electron Device Lett. 2013, 34 (2), 220–222. 10.1109/LED.2012.2229457.

[ref24] Hu P.; Hu P.; Vu T. D.; Li M.; Wang S.; Ke Y.; Zeng X.; Mai L.; Long Y. Vanadium Oxide: Phase Diagrams, Structures, Synthesis, and Applications. Chem. Rev. 2023, 123 (8), 4353–4415. 10.1021/acs.chemrev.2c00546. 36972332 PMC10141335

[ref25] Janod E.; Tranchant J.; Corraze B.; Querré M.; Stoliar P.; Rozenberg M.; Cren T.; Roditchev D.; Phuoc V. T.; Besland M.; Cario L. Resistive Switching in Mott Insulators and Correlated Systems. Adv. Funct Mater. 2015, 25 (40), 6287–6305. 10.1002/adfm.201500823.

[ref26] Yi W.; Tsang K. K.; Lam S. K.; Bai X.; Crowell J. A.; Flores E. A. Active Memristor Neurons for Neuromorphic Computing. In 2019 IEEE International Electron Devices Meeting (IEDM); IEEE, 2019; pp 22.7.1–22.7.4.

[ref27] Zaman A.; Shin E.; Yakopcic C.; Taha T. M.; Subramanyam G. Experimental Study of Memristors for Use in Neuromorphic Computing. In NAECON 2018 - IEEE National Aerospace and Electronics Conference; IEEE, 2018; pp 370–374.

[ref28] Fan L.; Chen Y.; Liu Q.; Chen S.; Zhu L.; Meng Q.; Wang B.; Zhang Q.; Ren H.; Zou C. Infrared Response and Optoelectronic Memory Device Fabrication Based on Epitaxial VO _2_ Film. ACS Appl. Mater. Interfaces 2016, 8 (48), 32971–32977. 10.1021/acsami.6b12831. 27934180

[ref29] Wu Z.; Shi P.; Xing R.; Yu T.; Zhao L.; Wei L.; Wang D.; Yan S.; Tian Y.; Bai L.; Chen Y. Flexible Mott Synaptic Transistor on Polyimide Substrate for Physical Neural Networks. Adv. Electron Mater. 2022, 8 (9), 2200078 10.1002/aelm.202200078.

[ref30] Darwish M.; Pohl L. Insulator Metal Transition-Based Selector in Crossbar Memory Arrays. Electronic Materials 2024, 5 (1), 17–29. 10.3390/electronicmat5010002.

[ref31] Dilna U.; Prasad S. N. An Efficient In-Memory Carry Select Adder Realization Using Resistive Switching Crossbar Array with Ti-Doped VO2 -Based Selector Device. Mater. Sci. Semicond Process 2024, 171, 108008 10.1016/j.mssp.2023.108008.

[ref32] Li Y.; Ang K.-W. Hardware Implementation of Neuromorphic Computing Using Large-Scale Memristor Crossbar Arrays. Advanced Intelligent Systems 2021, 3 (1), 2000137 10.1002/aisy.202000137.

[ref33] Ding G.; Zhao J.; Zhou K.; Zheng Q.; Han S.-T.; Peng X.; Zhou Y. Porous Crystalline Materials for Memories and Neuromorphic Computing Systems. Chem. Soc. Rev. 2023, 52 (20), 7071–7136. 10.1039/D3CS00259D. 37755573

[ref34] Zhou K.; Shang G.; Hsu H.; Han S.; Roy V. A. L.; Zhou Y. Emerging 2D Metal Oxides: From Synthesis to Device Integration. Adv. Mater. 2023, 35 (21), 2207774 10.1002/adma.202207774. 36333890

[ref35] Fairley N.; Fernandez V.; Richard-Plouet M.; Guillot-Deudon C.; Walton J.; Smith E.; Flahaut D.; Greiner M.; Biesinger M.; Tougaard S.; Morgan D.; Baltrusaitis J. Systematic and Collaborative Approach to Problem Solving Using X-Ray Photoelectron Spectroscopy. Applied Surface Science Advances 2021, 5, 100112 10.1016/j.apsadv.2021.100112.

[ref36] Ureña-Begara F.; Crunteanu A.; Raskin J.-P. Raman and XPS Characterization of Vanadium Oxide Thin Films with Temperature. Appl. Surf. Sci. 2017, 403, 717–727. 10.1016/j.apsusc.2017.01.160.

[ref37] Martinez J.; Dionizio S.; Gutierrez N.; Mosquera E.; Diosa J. E.; Bolaños G.; Moran O. General Aspects of the Physical Behavior of Polycrystalline BiFeO3/VO2 Bilayers Grown on Sapphire Substrates. Appl. Phys. A: Mater. Sci. Process. 2022, 128 (8), 720 10.1007/s00339-022-05798-1.

[ref38] Liu X.; Wang S.-W.; Chen F.; Yu L.; Chen X. Tuning Phase Transition Temperature of VO _2_ Thin Films by Annealing Atmosphere. J. Phys. D Appl. Phys. 2015, 48 (26), 265104 10.1088/0022-3727/48/26/265104.

[ref39] Manju; Jain M.; Madas S.; Vashishtha P.; Rajput P.; Gupta G.; Kahaly M. U.; Özdoğan K.; Vij A.; Thakur A. Oxygen Vacancies Induced Photoluminescence in SrZnO_2_ Nanophosphors Probed by Theoretical and Experimental Analysis. Sci. Rep 2020, 10 (1), 17364 10.1038/s41598-020-74436-8. 33060718 PMC7567121

[ref40] Ismail M. H.; Ali A. H.; Thahab S. M. Fabrication and Characterization of a VO2: PVP/PSi/ and n-Si Heterojunction for Photodetector Applications. Optics Continuum 2023, 2 (6), 1301 10.1364/OPTCON.484653.

[ref41] Mjejri I.; Etteyeb N.; Somrani S.; Sediri F. Tetragonal Pencil-like VO2(R) as Electrode Materials for High-Performance Redox Activities. Ceram. Int. 2016, 42 (5), 6121–6128. 10.1016/j.ceramint.2015.12.173.

[ref42] Simo A.; Beukes P.; Kaviyarasu K.; Numan N.; Fuku X.; Nkosi M. Structural and Luminescence Properties Changes Study of VO2 Nanostructures Induced by Gamma Irradiation. Mater. Today Proc. 2021, 36, 595–599. 10.1016/j.matpr.2020.05.651.

[ref43] Niu C.; et al. Synthesis and Optical Property of Size-Tunable Vanadiumoxide Nano-Dandelions. J. Nanosci. Lett. 2012, 3 (27), 1–4.

[ref44] Saini M.; Dehiya B. S.; Umar A.; Goyat M. S. Phase Modulation in Nanocrystalline Vanadium Di-Oxide (VO2) Nanostructures Using Citric Acid via One Pot Hydrothermal Method. Ceram. Int. 2019, 45 (15), 18452–18461. 10.1016/j.ceramint.2019.06.063.

[ref45] Xu J.; Hu C.; Xi Y.; Peng C.; Wan B.; He X. Synthesis, Photoluminescence and Magnetic Properties of Barium Vanadate Nanoflowers. Mater. Res. Bull. 2011, 46 (6), 946–950. 10.1016/j.materresbull.2011.02.023.

[ref46] Anpo M.; Tanahashi I.; Kubokawa Y. Photoluminescence and Photoreduction of Vanadium Pentoxide Supported on Porous Vycor Glass. J. Phys. Chem. 1980, 84 (25), 3440–3443. 10.1021/j100462a026.

[ref47] Joshi S.; Smieszek N.; Chakrapani V. Effects of Charge Fluctuation and Charge Regulation on the Phase Transitions in Stoichiometric VO2. Sci. Rep 2020, 10 (1), 17121 10.1038/s41598-020-73447-9. 33051507 PMC7553960

[ref48] Xiao M.; Musselman K. P.; Duley W. W.; Zhou Y. N. Reliable and Low-Power Multilevel Resistive Switching in TiO _2_ Nanorod Arrays Structured with a TiO _*x*_ Seed Layer. ACS Appl. Mater. Interfaces 2017, 9 (5), 4808–4817. 10.1021/acsami.6b14206. 28098978

[ref49] Tang J.; Yuan F.; Shen X.; Wang Z.; Rao M.; He Y.; Sun Y.; Li X.; Zhang W.; Li Y.; Gao B.; Qian H.; Bi G.; Song S.; Yang J. J.; Wu H. Bridging Biological and Artificial Neural Networks with Emerging Neuromorphic Devices: Fundamentals, Progress, and Challenges. Adv. Mater. 2019, 31 (49), 1902761 10.1002/adma.201902761. 31550405

[ref50] Subin P. S.; Asha A. S.; Saji K. J.; Jayaraj M. K. Spike-Dependent Plasticity Modulation in TiO2-Based Synaptic Device. Journal of Materials Science: Materials in Electronics 2021, 32 (10), 13051–13061. 10.1007/s10854-021-05710-2.

[ref51] Guo J.; Liu L.; Bian B.; Wang J.; Zhao X.; Zhang Y.; Yan Y. Ligand Exchange Reaction Enables Digital-To-Analog Resistive Switching and Artificial Synapse within Metal Nanoparticles. Adv. Funct Mater. 2023, 33 (16), 2212666 10.1002/adfm.202212666.

[ref52] Wu Y.; Huang H.; Xu C.; Cao X.; Lei Z.; Zhang J.; Zhao Y.; Wei A.; Liu Z. The FAPbI3 Perovskite Memristor with a PMMA Passivation Layer as an Artificial Synapse. Appl. Phys. A: Mater. Sci. Process. 2023, 129 (5), 364 10.1007/s00339-023-06632-y.

[ref53] Zhang X.; Liu S.; Zhao X.; Wu F.; Wu Q.; Wang W.; Cao R.; Fang Y.; Lv H.; Long S.; Liu Q.; Liu M. Emulating Short-Term and Long-Term Plasticity of Bio-Synapse Based on Cu/a-Si/Pt Memristor. IEEE Electron Device Lett. 2017, 38 ( (9), ). 1208 10.1109/LED.2017.2722463.

[ref54] Hu S. G.; Liu Y.; Chen T. P.; Liu Z.; Yu Q.; Deng L. J.; Yin Y.; Hosaka S. Emulating the Paired-Pulse Facilitation of a Biological Synapse with a NiOx-Based Memristor. Appl. Phys. Lett. 2013, 102 (18), 183510 10.1063/1.4804374.

[ref55] Tanim M. M. H.; Templin Z.; Zhao F. Natural Organic Materials Based Memristors and Transistors for Artificial Synaptic Devices in Sustainable Neuromorphic Computing Systems. Micromachines (Basel) 2023, 14 (2), 235 10.3390/mi14020235. 36837935 PMC9963886

[ref56] Hu S. G.; Liu Y.; Chen T. P.; Liu Z.; Yang M.; Yu Q.; Fung S. Effect of Heat Diffusion During State Transitions in Resistive Switching Memory Device Based on Nickel-Rich Nickel Oxide Film. IEEE Trans. Electron Devices 2012, 59 (5), 1558–1562. 10.1109/TED.2012.2186300.

[ref57] Lee M.-J.; Han S.; Jeon S. H.; Park B. H.; Kang B. S.; Ahn S.-E.; Kim K. H.; Lee C. B.; Kim C. J.; Yoo I.-K.; Seo D. H.; Li X.-S.; Park J.-B.; Lee J.-H.; Park Y. Electrical Manipulation of Nanofilaments in Transition-Metal Oxides for Resistance-Based Memory. Nano Lett. 2009, 9 (4), 1476–1481. 10.1021/nl803387q. 19296606

[ref58] Ahmed T.; Walia S.; Mayes E. L. H.; Ramanathan R.; Bansal V.; Bhaskaran M.; Sriram S.; Kavehei O. Time and Rate Dependent Synaptic Learning in Neuro-Mimicking Resistive Memories. Sci. Rep 2019, 9 (1), 15404 10.1038/s41598-019-51700-0. 31659247 PMC6817848

[ref59] Serrano-Gotarredona T.; Masquelier T.; Prodromakis T.; Indiveri G.; Linares-Barranco B. STDP and STDP Variations with Memristors for Spiking Neuromorphic Learning Systems. Front. Neurosci. 2013, 7, 2 10.3389/fnins.2013.00002. 23423540 PMC3575074

[ref60] Shuai Y.; Pan X.; Sun X. Spike-Timing-Dependent Plasticity in Memristors. In Memristor and Memristive Neural Networks; InTech, 2018.

[ref61] Babacan Y.; Kaçar F. Memristor Emulator with Spike-Timing-Dependent-Plasticity. AEU - International Journal of Electronics and Communications 2017, 73, 16–22. 10.1016/j.aeue.2016.12.025.

